# Suicide prediction, prevention, and the blame culture: A narrative review based on trends in mortality rates

**DOI:** 10.1192/j.eurpsy.2025.10139

**Published:** 2025-11-24

**Authors:** Julian Beezhold, Tommy Porter, Mariana Pinto da Costa, Jan Wise, Asilay Seker, Martina Rojnic-Kuzman, Livia de Picker, Victoria Selwyn, Victor Buwalda, Defne Eraslan, Cecile Hanon, Rose Winter, Michael Briggs, Sarah Heales, Toby Whitelock, Dawn Collins

**Affiliations:** 1 Norwich Medical School, University of East Anglia, Norwich, UK; 2 UEA: University of East Anglia, UK; 3 Institute of Psychiatry at the Maudsley: King’s College London Institute of Psyc, UK; 4 Central and North West London NHS Foundation Trust, UK; 5 University of Zagreb Faculty of Medicine: Sveuciliste u Zagrebu Medicinski Fakul, Croatia; 6 Universiteit Antwerpen, Belgium; 7 Hertfordshire Partnership NHS Foundation Trust: Hatfield, Hertfordshire, UK; 8 GGD Amsterdam, Netherlands; 9 SRH Klinikum Karlsbad-Langensteinbach GmbH, Germany; 10 Hopital Corentin Celton, France; 11 Norfolk and Suffolk NHS Foundation Trust, UK

**Keywords:** blame culture, suicide, suicide investigations, suicide mortality rates, suicide prediction, suicide prevention

## Abstract

**Background:**

Suicide involves an act of volition on the part of the deceased, making it unlike deaths from physical disorders such as cancer or stroke. The latter occur passively and often despite the efforts of the patient to stay alive. Yet when there is a suicide, clinicians involved may often be blamed and families may often feel guilt. This contrasts with the default response of praise and thanks to clinicians following treatment preceding deaths from physical disorders.

**Methods:**

Comparative standardized mortality rate (SMR) data are analyzed to demonstrate the impact of developments in care over the past two decades in the United Kingdom (UK), and similar United States (USA) SMR data are noted. The evidence is reviewed regarding our ability to predict who will die by suicide, when and where to target intervention, and practical and effective prevention methods.

**Results:**

Data from the UK are presented that reflects the relative lack of impact of prevention efforts on suicide mortality rates when compared to the reductions seen in various physical disorders. This narrative review comments on the causes and consequences of this difference.

**Conclusions:**

The challenge for psychiatry is that SMR data suggest that we have been unable to significantly reduce suicide SMR unlike that for physical disorders. This needs to be fully acknowledged and the biased assumption of blame needs to stop. The focus needs to be on evidence-based interventions that do work, such as medications, psychological treatments, psychological interventions, and suicide prevention research.

## Introduction

Increased understanding of data regarding suicide mortality trends may contribute to a better understanding of suicide prevention challenges and help a move away from the present culture of assumed blame [1].

Successful suicide prevention requires that we can accurately identify those most at risk, understand when and how to effectively intervene, and have interventions that work. Yet Tom Insel, former National Institute for Mental Health (NIMH) Director, has suggested that our efforts in this regard appear to have had little effect on mortality rates for suicide when compared to rates for physical disorders [2].

### Suicide and the culture of blame

There are two aspects of suicide that make it fundamentally different from all other causes of death. First, suicide is, by definition, an intentional act on the part of the deceased [3, 4]. It almost always happens despite attempts to help the person. Second, there is a default assumption that deficient care must be to blame, or conversely, that better care would have prevented the suicide. This second assumption is very often internalized, with both family and clinicians often feeling that they must have done something wrong or should have done better.

This can feed into the deeply stigmatizing blame culture in which society’s understanding of suicide is situated, and which contributes to the devastating emotional impact on family and friends [1, 5]. The odds ratio (OR) for further suicides in direct family members after a death by suicide is up to three times higher than for the general population [6, 7] with the emotional impact of a loved one’s suicide a potential trigger.

For clinicians, a death from, for example, diabetes would generally tend to be perceived by family, fellow clinicians, and the public as having taken place despite all efforts to help the patient. Yet the perception with suicide is the exact opposite, being one of blame and suspicion that the patient must have been somehow failed by clinicians and services [8, 9].

This assumption may be externalized through investigations that almost always recommend changes – with the clear imputation that there were blameworthy failures in care, without which the suicide would not have happened. Yet the investigations themselves have been criticized as flawed, with frequent hindsight bias, and not fit for purpose [10–12].

The stress of being under investigation, regardless of the outcome, can have severe consequences for clinicians, and is compounded by the potentially flawed investigative process.

An Internal Review was conducted of 114 doctors who died during the period 2005–2013 while under investigation by the UK General Medical Council (GMC). It found that 28 (24.5%) of these were due to suicide or suspected suicide [13, 14]. These findings reflect an association rather than causation. However, to put this in perspective, the proportion of suicides by doctors while under GMC investigation was more than 24 times higher than that for suicides in the general population, which account for about 1% of all deaths in the United Kingdom and worldwide [15].

The same GMC review stated: *“Often a doctor will be involved in a number of investigation processes at the same time (multiple jeopardy), with the complaint process being stressful; the nature of multiple investigations including employer disciplinary processes means investigations can take many years, be intimidating and can lead to mental health problems and even suicide”* [14].

Finally, this blame culture is further externalized in broader society by print, broadcast, and social media reporting and portrayal, which inform and underpin public understanding and expectations [1]. The rise of social media presents many new challenges due to the generally low level of content moderation. Harsh criticism, accusations, misinformation, and the lure of sensationalism may all magnify blaming in case of death by suicide [16].

The World Health Organization and others have produced guidance for media producers to reduce harm from media discussion of suicide with some signs that there has been improvement in print and broadcast media [17].

Multiple investigations by different authorities may follow a single suicide – see, for example, Table 1. These may further reinforce the blame culture by creating the perception that when there is a suicide then there must have been a failure of care.

**Table 1.** Possible investigations following a single suicide in England

Internal
Serious incident investigation
Quality and safety review
Complaint investigations
Disciplinary investigations
External
Independent external investigation
Police
Health service ombudsman
Regulatory bodies for professional staff (e.g. General Medical Council)
Medical practitioners tribunal service
Professional standards authority
Parliamentary inquiries which may have judicial powers
Coroners’ courts
Civil courts
Criminal courts
Care quality commission
Clinical commissioning group
Local council health overview and scrutiny committee
NHS Resolution Practitioner Performance Advice Service
Doctors in training may further be investigated by training authorities

This blame culture in the United Kingdom and United States has strikingly failed to reduce the suicide rate compared to the advances in many physical disorders – and the time has come for a wiser, more humane, and less stigmatizing approach based on evidence and focused more on improving the quality of care for those with mental disorders.

## Methods

This narrative review analyses UK government data on the standardized mortality rates (SMRs) for suicide and selected physical disorders and notes similar USA findings.

The societal prejudice that suicide must be due to poor care, unlike, for example, cancer deaths, is examined in light of the available evidence regarding our (in)ability to predict suicide in an individual, and the challenges of providing effective preventive interventions to the right person at the right time.

The reasons for this failure to reduce the suicide SMR are examined by considering the following practical challenges for successful prevention of suicide include: (1) Can we identify those who should receive suicide-prevention intervention? (2) Can we identify the best time for delivery of a suicide-prevention intervention for potentially suicidal individuals? (3) Do we have effective clinical interventions, that are practical and achievable, that would prevent suicides or at least significantly reduce the risk?

## Results

### Can we identify those who should receive suicide-prevention intervention?

We know that, for example, in the United Kingdom, about 75% of those who die by suicide were not under the care of mental health services at the time of their death [18]. A reasonable inference from this may be that society, and particularly those closest to the person who died by suicide, were unaware of increased concerns sufficient to prompt referral to mental health services. This is consistent with the hypothesis that it is very difficult to accurately identify whether somebody is going to die by suicide. Much research has been done regarding people in contact with mental health services to identify those most at risk of suicide.

This has identified many factors that are associated with an increased risk of suicide [18–21].

Risk factors for future suicide are generally formulated as either being associated with a lifetime risk of suicide [19] or with a risk of suicide within the following year [20].

Windfuhr and Kapur [18], in a review of review of 15 years of UK National Confidential Inquiry into Suicide findings, found that an increased risk of suicide is related strongly to the presence of mental disorder; advancing age; and to gender, with males significantly more likely to die by suicide. This is consistent with World Health Organization data [21].

A systematic review and meta-analysis of psychological autopsy studies [19] found that the highest OR of >10 was for those receiving psychiatric treatment, those with a previous history of self-harm, and those with an adverse life-event within the previous month. They found that the following are all associated with an OR for suicide of >5: family history of mental disorder, depression, schizophrenia, borderline/paranoid/dependent personality disorders, receipt of psychiatric treatment, previous self-harm, previous suicide attempt, and relationship conflict.

A large meta-review of suicide in people with mental disorders [22] found that “*for suicide mortality, borderline personality disorder, depression, bipolar disorder, opioid use, and schizophrenia, as well as anorexia nervosa and alcohol use disorder in women, had substantially increased rates (greater than 10 times) compared with the general population.”*

Ryan et al. [23] stated in an editorial that *“Patients who present in psychological crisis or after a suicide attempt are more than 50 times more likely than the general population to die by suicide in the following year.”* This has been confirmed in multiple studies, such as a retrospective cohort study of 3987 Korean patients by Choi et al. [24] and a prospective cohort study of 29,571 patients by Steeg et al. [25].

It appears intuitively that the best opportunity for intervention would be with those at the highest risk of suicide.

Many forms of suicide risk assessment have been developed to identify those at highest risk. Examples include the Patient Safety Screener-3 [26], the Pediatric Emergency Care Applied Research Network Computerized Adaptive Screen for Suicidal Youth [27], Columbia-Suicide Severity Rating Scale (C-SSRS) [28], Ask Suicide-Screening Questions [29], and the Beck Scale for Suicide Ideation [30].

Unfortunately, there is no known suicide risk assessment that has been shown to effectively identify those individuals who will die by suicide and thereby target interventions and prevent suicides.

There are several reasons for this. At the time of any risk assessment, most people who go on to die by the intentional act of suicide will not yet have made the suicide decision [31–35]. If they themselves do not yet know, then it is presumably difficult for a clinician to discover what that person’s future decision will be. Therefore, clinicians are guided by the suicide risk factors identified and by their clinical assessment of the individual.

Suicide rates are generally low, with age-standardized annual rates ranging worldwide between 0.31 and 87.48 according to 2019 World Health Organization [36]. The rate in most of Europe, Africa, Asia, the Americas, and Australia is less than 15/100,000/year.

The low population base rate of suicide leads to risk categorization methods identifying many more people as being at the very highest risk than will die by suicide in the following year. Steeg et al. [25], in a multicenter prospective cohort study of 29,571 self-harm presentations, found: “Although patients presenting after suicide attempts or in crisis have a greatly increased probability of eventual suicide compared with the general community, fewer than one in 200 will actually die by suicide in the next 6 months.”

Madsen et al. [37], in a Danish national, prospective, register-based study, found “that only 12% of suicides occurred among high-risk patients, and that fewer than one in 400 high-risk patients died by suicide.”

The clinical implication is that the best possible system of risk assessment, looking at the highest risk group of psychiatric inpatients, would identify as the very “highest risk” group about 98 people who will, in fact, not die by suicide in the next year for every two who do; and, even more frustratingly, would miss the majority of those who do go on to die by suicide because they would be assessed as being at “low” risk [38, 39].

This was looked at in detail in a systematic review and meta-analysis by Large: “Even making assumptions that optimize the power of suicide risk assessment, we estimated that while some (in)patients could be categorized as “ultra-high-risk,” as few as 3% of these would go on to suicide in the year after discharge. Meanwhile, about 60% of people who did eventually suicide during the same period would have been categorized as being at lower risk.” “Despite the apparently strong association between high-risk categorization and subsequent suicide, the low base rate of inpatient suicide means that predictive value of a high-risk categorization is below 2%” [38].

In the United Kingdom, the National Institute of Health and Care Excellence has acknowledged the reality that suicide risk assessment tools are not clinically useful by recommending:

*“1.6 Risk assessment tools and scales*

*1.6.1 Do not use risk assessment tools and scales to predict future suicide or repetition of self-harm.*

*1.6.2 Do not use risk assessment tools and scales to determine who should and should not be offered treatment or who should be discharged.*

*1.6.3 Do not use global risk stratification into low, medium or high risk to predict future suicide or repetition of self-harm.*

*1.6.*4 *Do not use global risk stratification into low, medium or high risk to determine who should be offered treatment or who should be discharged.”* [40]

The challenge for suicide prevention is that, despite all efforts, no tool or method has been found to identify those who will go on to die by suicide with clinically useful accuracy. The presence of suicide risk factors cannot tell us whether or when an individual may attempt suicide [38–42].

Studies, using blind ratings of suicide risk by case note review, appear to confirm this inability to usefully predict suicide and the danger in investigations of hindsight bias [43, 44].

### Can we identify the best time for delivery of a suicide-prevention intervention for potentially suicidal individuals?

Common sense suggests that suicide-prevention interventions, that are delivered after the decision to die by suicide has been taken, may most likely succeed in preventing the death. Examples include talking down a person about to jump from a high building or treating for an overdose.

Unfortunately, research (Table 2) suggests that for very many people who attempt suicide, there is often a period of only minutes between the decision and the act [31–35]. This means there would typically be a very brief window of opportunity for intervention. A clinician would have to discover the suicide decision during this brief window to act. Realistically, this makes intervention at the right time very challenging and often impossible to achieve.

**Table 2.** Suicide: Time from the decision taken, to the suicidal act made

	<5 min	<10 min	<30 min	<1 h
Williams et al. [31]	40%			
Simon et al. [32]	24%			71%
Eddleston et al. [33]			50%	
Deisenhammer et al. [34]		48%		
Paashaus et al. [35]	36%	44%		

There currently appears to be no practical way of identifying when those most at risk should be targeted for intervention. The typically very short window of opportunity to intervene with suicide prevention between the suicide decision and the suicide act adds considerably to this difficulty. Clinical staff would need to be in the right place at the right time to successfully intervene, which is unfeasible given the very small proportion of those deemed at highest risk who go on to die by suicide. Put differently, a flawless suicide-prevention service would have to treat 100 of the highest risk patients for a whole year with perfect treatment to prevent two or three suicides [25, 37, 38].

### Do we have effective clinical interventions, that are practical and achievable, that would prevent suicides or at least significantly reduce the risk?

There are a multitude of care initiatives and clinical interventions that are aimed at preventing suicide, with many publications showing effectiveness at reducing suicide rates [44–46].

Hawton and Sinyor commented in an editorial on the challenge of showing whether they work:

*“It is difficult to demonstrate that reductions in suicide rates seen in most countries reflect the introduction of national suicide prevention strategies. However, there is evidence regarding some of the specific initiatives that often comprise such strategies, including what is effective and what seems not to work. The suicide prevention field is also rapidly evolving, with many promising approaches still needing fuller evidence.”* [46]

Various environmental interventions have played a role in different parts of the world. Multiple methods of restricting access to means of suicide have shown evidence of effectiveness. In the United States, increased access to guns has been associated with higher suicide rates, and decreasing gun access has been associated with an overall reduction in suicides [47, 48].

In the United Kingdom, coal gas was previously used as fuel for domestic cooking and heating. This was highly toxic because of the high levels of carbon monoxide produced during combustion, leading to a high number of suicides by inhalation. When coal gas was replaced with less toxic natural gas between the 1950s and 1970s, this change was associated with an overall reduction in the suicide rate [49].

A large international systematic review of multiple different means-restriction interventions found short-term benefit in some studies, but did not examine whether this effect was sustained in the long term [50].

A Finnish study using 43 years of suicide data found that multiple means restrictions were gradually followed by an increased use of other methods [51].

Admission to a psychiatric hospital is often used to try to prevent suicide. Some research evidence suggests that inpatient admission itself may not prevent suicide. [52] Evidence suggesting a lack of efficacy of admission in preventing suicide includes a natural experiment in Fulton County, Georgia, USA, when admission to a psychiatric hospital was cut by 56% due to reduced funding. During this period, the suicide rate fell from 12 to 10 per 100,000 population [53]. There is debate about whether admission is protective or may increase suicide risk. [54]

Other individual-level approaches include treating associated conditions, such as depression, with medications, cognitive behavioral therapy (CBT), dialectical behavioral therapy (DBT), and collaboratively developing a safety plan [55]. A systematic review of evidence-based suicide prevention [56] found evidence for training of primary-care physicians in depression recognition and treatment, and for means restriction such as firearms controls in the prevention of suicide. Educating youth regarding depression and suicidal behavior, active mental health service outreach postdischarge and post-self-harm, antidepressants, ketamine, and CBT and DBT were all found to reduce suicidal behaviors [55, 56]. There is some evidence suggesting that clozapine may have a protective effect [57].

Multimethod interventions have been proposed as a more effective way of preventing suicide, for example, the “Zero Suicide Model” [58] and “Alliances against Depression” [59], but there is an absence of convincing evidence of clinical efficacy [60], and criticism of the ethical [61] and research [62] consequences.

The published evidence, usually based on associations, suggests that many of these interventions prevent suicides [45, 46, 56]. Methodological issues include defining and separating outcome measures of suicidal ideation, suicidal behaviors with or without intent, and suicide itself [51]. The challenges in conducting gold-standard double-blinded, randomized controlled trials of these interventions means proving the cause–effect relationship is difficult, with a lack of robust evidence of sustained efficacy [63, 64].

Unfortunately, there is little evidence of a significant reduction in population-level mortality that might have been expected to flow from these promising interventions.

In the United Kingdom, suicide rates have not dropped since the National Confidential Inquiry into Suicide and Homicide (NCISH) was established in 1996. The suicide mortality rate was 10.9/100,000 in 1996 and slightly higher 11.4/100,000 in 2023 [65].

In contrast, deaths in surgery dropped initially, following into Peri-Operative Death from 121/10,000 in 1998 to 76/10,000 in 2014 [66]. Following the Confidential Enquiry into Maternity Services Deaths mortality rates dropped from 90/100,000 in 1952 to 8.79/100,000 in 2017–2019) [67].

Encouraging results from suicide prevention research and interventions have been described [45, 46, 56]. However, these have had comparatively little impact on the overall suicide mortality rate when compared to the reductions in those for physical disorders.

This is revealed by an examination of national population mortality rate trends, for example, in England [68] and the United States [2], where there is high-quality mortality data available.

In England [68] and the United States [2], there has been an often dramatic improvement in mortality rates for most medical conditions over the past couple of decades. Major decreases have been seen, for example, in the SMR for heart disease and cancers.

**Figure 1.**
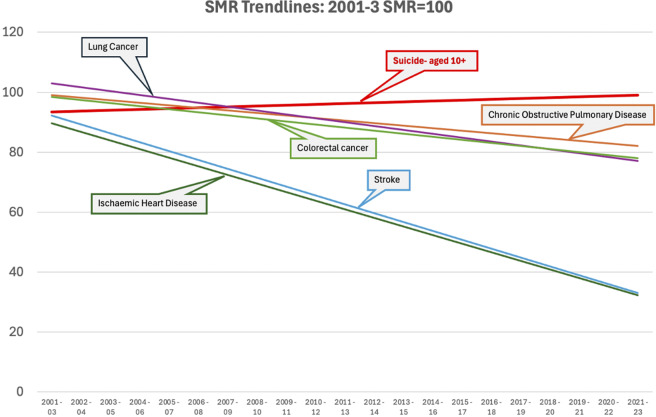
Changes in mortality rates – England [68].

Yet this is not true of suicide. The suicide mortality rate has not reduced in the way it has for other conditions, as illustrated in Figures 1 (England) and 2 (United States). The many different interventions, past and present, including initiatives to improve medications, psychological therapies, and in-/out-patient care have not led to a similar meaningful reduction in overall suicide rates.

**Figure 2.**
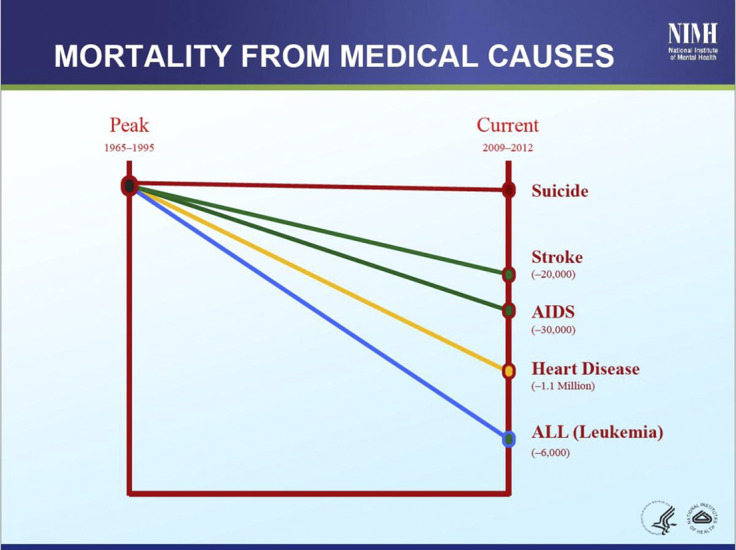
Changes in mortality rates – United States (Insel/NIMH) [2].

Figure 1 shows data for England illustrating the comparative trend from 2001 to 2023 in SMR for suicide compared to colorectal cancer, lung cancer, chronic obstructive pulmonary disease, stroke, and ischemic heart disease [68].

Tom Insel, then Director of the United States NIMH, pointed out what he called the “*inconvenient truth*” that all the efforts of mental health clinicians and researchers have essentially had little impact on suicide mortality compared to some other conditions. He infers from this that psychiatry has not yet discovered how to prevent suicide [2].

Mortality rate trends in England for several physical disorders show major reductions over recent decades. However, these data (Figure 2) reveal that despite all our efforts, all the research, and all the clinical and environmental interventions, there is little evidence of therapeutic interventions that have similarly reduced suicide mortality rates.

This illustrates how hard it is to prevent suicide. The assumption that suicide can be systematically prevented if only we identify and treat people who may suicide is therefore not supported by the available research evidence.

### Do health services correctly target those most at risk of suicide?

We should consider whether mental health services correctly target those who are at highest suicide risk.

More intensive treatment is associated with higher rates of suicide, suggesting that healthcare is to some extent correctly targeted at those most at risk. Hjorthøj et al. [20], in a nested case–control study of 2429 cases and 50,323 controls in Denmark, found that the more intensive the treatment, the higher the suicide risk in the following year. This association of higher levels of care with higher suicide risk appears to suggest that health services do, to some extent, target those at higher suicide risk.

The UK NCISH found that the risk of suicide after hospital discharge is at its highest in the first days after discharge and then decreases day-by-day thereafter [69]. While these data cannot show causation, it appears to be consistent with the hypothesis that patients are generally discharged at a time when their suicide risk is reducing.

Despite this, there has been no reduction in the overall suicide mortality rate.

Counterintuitively, and without evidence of causality, some even infer that the treatments themselves cause suicide, for example, antidepressant medication [70, 71].

## Discussion

Recognition of these real-life clinical challenges, and of ending the blame culture, is crucial for clinicians, but most of all for bereaved family and friends who for too long have had to bear this unjustified double burden of bereavement and (self) blame. We, as a society, need to avoid colluding with this false narrative of blame.

Careful consideration needs to be given to how suicide-related investigations are conducted given the dangers of hindsight bias in the context of a well-intentioned but misguided blame culture.

Real-world examples suggest that changing the blame culture is very difficult. In the United Kingdom, initiatives such as the NCISH; the “*Learning, Not Blaming”* policy 2015 [72], the establishment of the Health Service Safety Investigation Branch in 2016, which became the Health Services Safety Investigations Body in 2023, and the National Quality Board that introduced new guidance “*National Guidance on Learning from Deaths*” [73], appear to have not made much difference to suicide mortality rates.

This blame culture is reflected in the key messages page of the UK 2024 NCISH Annual Report webpage that states [74]:

*“There is concern currently about safety in mental health in-patient services. During 2011-2021, over a quarter (28%) of patients died by suicide in acute mental health care settings, including in-patients (6%), post-discharge care (14%) and crisis resolution/home treatment (13%). Of the estimated 74 suicides by mental health in-patients in the UK, a quarter (28%) died whilst under enhanced nursing observation (i.e., frequent – every 15-30 minutes – checks on a patient or being with them constantly). The highest number of deaths after discharge from psychiatric in-patient care occurred on day three post-discharge.*

*We recommend clinical services need to focus on (1) creating a therapeutic ward environment, (2) the physical safety of the in-patient unit itself, (3) safe transition from ward to community, (4) pre- and post-discharge, (5) early follow-up after in-patient discharge, and (6) prompt access to crisis services.”* [74]

Aside from the incorrect statement (presumably unintentionally) suggesting that 28% of acute inpatients died by suicide, simply saying things are important does not mean that implementation changes the suicide rate. Another way of understanding the same figures is that they illustrate just how difficult it is, despite all our efforts, to prevent suicides. The report implies that suicides represent a failure of care, due to insufficient “focus” on the six recommendations, despite there being no evidence that action on these recommendations significantly reduces suicide mortality.

### What can be done now?

Using suicide as an outcome measure for mental health care appears well-intentioned and common sense. However, the causes of suicide are multifactorial and complex. A mental disorder and subsequent suicide are not necessarily cause and effect in the same way as in other medical conditions, for example, leukemia and subsequent death. Treating one potential issue does not necessarily mean treating the cause of a potential suicide. Fundamentally, suicide is an intentional act on the part of the individual, whereas deaths from other medical disorders result passively from lethal illness progression.

Ongoing work to improve the quality of mental health care continues to be needed. It is a challenge for all of us to secure appropriate, significantly increased investment and funding for the major unmet needs in mental health care and research.

Most important is optimizing the care provided for people with mental disorders. Unlike suicide prevention, there is an abundance of evidence for many effective pharmacological [75], psychological [76], social [77], and public health [78] interventions that work for mental disorders and make a real difference to people’s lives.

Patients, families, and carers should be fully involved in helping plan, shape, and deliver improvements and initiatives to reduce suicide. This will include building on the existing evidence base for effective prevention of suicide.

The reduction of suicide rates remains an important but elusive target and requires significant investment in methodologically robust research.

## Supporting information

10.1192/j.eurpsy.2025.10139.sm001 Beezhold et al. supplementary material Beezhold et al. supplementary material

## Data Availability

The data used for our analysis regarding Standardized Mortality Rates in England is publicly available on “United Kingdom Department of Health and Social Care, Fingertips | Public health profiles, Directly standardized mortality rates data” https://fingertips.phe.org.uk/profile/mortality-profile (accessed 30 December 2024). The data from these sources as extracted and used in our paper is available in tabular form as Supplementary Material.

## Supplementary material

The supplementary material for this article can be found at http://doi.org/10.1192/j.eurpsy.2025.10139.
