# Supplementary figures and images for: Suicide prediction, prevention, and the blame culture: A narrative review based on trends in mortality rates
Source: Eur Psychiatry. 2025 Nov 24;68(1):e172. doi: 10.1192/j.eurpsy.2025.10139 (PMC12721991; doi:10.1192/j.eurpsy.2025.10139)

**Supplementary materials:**

**Raw data for United Kingdom causes of death SMR trends [68]**


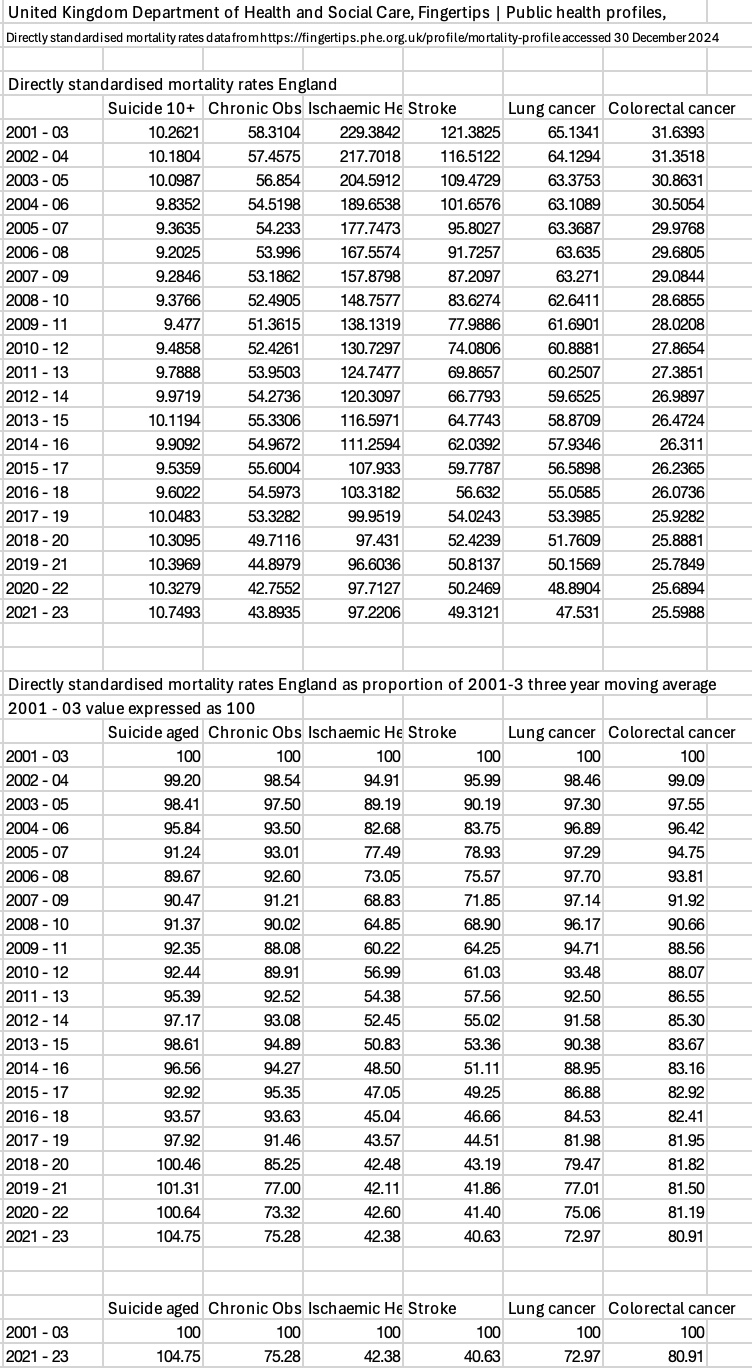

Supplement: Beezhold et al. supplementary material [file S0924933825101399sup001.docx]
